# Increased CO_2_ levels in the operating room correlate with the number of healthcare workers present: an imperative for intentional crowd control

**DOI:** 10.1186/s13037-022-00343-8

**Published:** 2022-11-17

**Authors:** Gregory T. Carroll, David L. Kirschman, Angela Mammana

**Affiliations:** 1 Aerobiotix, Inc, 444 Alexandersville Rd, 45342 Miamisburg, OH USA; 2 Department of Chemistry, University of Dayton, 300 College Park Dr, 45469 Dayton, OH USA

**Keywords:** CO_2_, Operating room, Surgical Site infection, Air Quality, Indoor air, Monitoring

## Abstract

The air in an operating room becomes more contaminated as the occupancy of the room increases. Individuals residing in a room can potentially emit infectious agents. In order to inhibit and better understand the epidemiology of surgical site infections, it is important to develop procedures to track room occupancy level and respiration. Exhaled CO_2_ provides a respiratory byproduct that can be tracked with IR light and is associated with human occupancy. Exhaled CO_2_ can also be used as an indirect measure of the potential release and level of infectious airborne agents. We show that non-dispersive infrared CO_2_ sensors can be used to detect CO_2_ in operating room air flow conditions of 20 air changes per hour and a positive pressure of 0.03 in. H_2_O. The CO_2_ concentration increased consecutively for occupation levels of one to four individuals, from approximately 65 ppm above the background level when one individual occupied the operating room for twenty minutes to approximately 300 ppm above the background when four individuals were present for twenty minutes. The amount of CO_2_ detected increases as the number of occupants increase, the activity level increases, the residency time increases and when the ventilation level is reduced.

**Supplementary Information:**

The online version contains supplementary material available at 10.1186/s13037-022-00343-8.

## Introduction

**Fig. 1:**
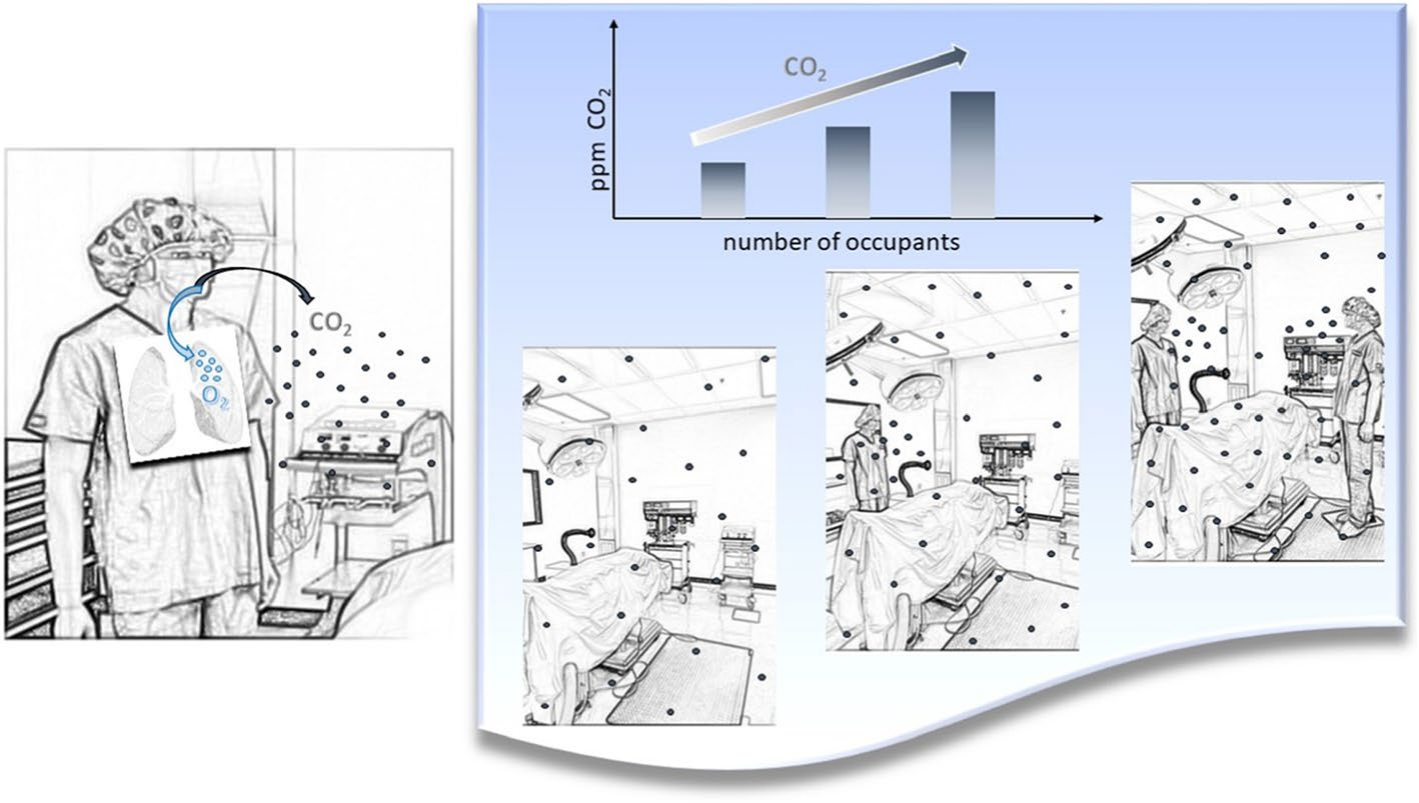
Exhalation produces CO_2_. CO_2_ accumulates in the air of an occupied operating room. As more individuals populate the room, the rate of CO_2_ accumulation in the room increases

In-door air quality is affected by a variety of factors including human occupation [1–3]. Developing and applying effective and non-invasive methods that monitor the amount of people in a room is particularly important in clinical settings where air quality is a major factor that can contribute to nosocomial infection including surgical site infections [4, 5]. It has been reported that 2.0-2.4% of all total joint arthroplasties in the U.S. result in periprosthetic infection [6], which imposes significant morbidity on patients and large financial burdens on the healthcare system [7], making the pursuit of improved air quality in operating rooms of high importance. In general, the quality of the air in an operating room (OR) will be compromised by various viable and non-viable particulate matter and small molecules as the number of people in the room increases [8]. For example, bioaerosols are emitted into the surrounding environment as people breathe, talk, laugh, cough or sneeze [9]. Additionally, human skin sheds squamous epithelial cells which can contain micro-organisms [10]. A promising strategy for enhancing OR environment management involves the application of automated sensors to track the flow of human traffic and respiration over time. It is well-known that occupants contribute to the concentration of indoor CO_2_ [11–13]. Carbon dioxide (CO_2_) provides a detectable marker of respiration that can be routinely monitored and is expected to increase in concentration in an indoor environment as more individuals occupy a room or building (Fig. 1). CO_2_ emissions from individuals under various activity levels and environmental conditions is an active area of research. Recent studies have investigated the effect of various conditions including sedentary vs active behavior, indoor pollutants, ambient CO_2_ levels, stressed vs relaxed cognitive activities, metabolic rate, temperature, gender and age [14–21]. Measurement of CO_2_ concentration has previously been suggested as a tool for estimating airborne infection transmission risk [22]. Recently, analytical expressions were derived for the probability of Covid-19 infection based on CO_2_ levels in a number of indoor environments [23]. However, little data is available on the behavior and detectability of occupant-driven CO_2_ levels in the operating room environment.

CO_2_ exists in a gaseous state under ambient conditions and is a by-product of respiration. The respiration rate of a healthy adult is approximately 14-18 respirations per minute and the tidal volume of air associated with breathing is approximately 0.5 L [24]. Exhalation expels CO_2_ in an amount that is approximately 5% of the total volume of gas released. The amount of CO_2_ expelled by a healthy adult at rest is approximately 0.35–0.45 L/min. On average, an adult will release about 1.0 kg (580 L at 1 atm and 25º C) of CO_2_ per day. CO_2_ is generated during metabolism at a rate that is dependent on the physical activity of the individual. Higher levels of exhaled CO_2_ are associated with the production of higher levels of bioaerosols as both are byproducts of breathing. Heightened levels of bioaerosols increase the risk of infection for both patients and staff in an OR. High levels of CO_2_ in a room are also associated with discomfort and reduced cognitive abilities [25–29]. Understanding how the level of CO_2_ correlates with the number of occupants in an OR and incorporating CO_2_ sensing devices into clinical settings will provide significant insight into assessing factors that lead to post-operative wound infections [30].

CO_2_ detectors are readily available from a large number of manufacturers. While they have been used in a variety of environments, their utility in an OR would be clearer if carefully assessed in a controlled OR environment. Electrochemical gas sensors are subject to false readings from interfering gases. In comparison, NDIR (non-dispersive infrared) gas sensors [31, 32] are advantageous in that the readings are not affected by the presence of other gases as the characteristic IR absorption band of CO_2_ at approximately 2350 cm^-1^ (4.26 mm) does not overlap with common gases. Note that CO shows absorption maxima between 2100 and 2200 cm^-1^, which provides sufficient spacing to distinguish CO_2_ from CO. Additionally, NDIR sensors last longer and do not lose accuracy over time, whereas electrochemical sensors have a reduced shelf-life and wear out more quickly with usage as the electrolyte is consumed. Temperature and humidity can also reduce an electrochemical sensor’s lifetime. Importantly, NDIR sensors can measure CO_2_ concentrations below the ambient outdoor level of 400 ppm, whereas metal-oxide semiconductor-based sensors (a type of commercially available electrochemical sensor) generally have a lower limit of 2000 ppm, which is well above common ambient levels and therefore likely not suitable for measuring room occupation when few people are present [33]. While photoacoustic IR sensors that utilize micro-electromechanical systems technology appear to be exceptionally reliable, they are expensive relative to NDIR sensors and can cost over $40000 for one unit.

In this study, we examine the behavior of CO_2_ levels relative to the number of people and activity level in a simulated OR environment. We show that as the number of people in the room increases, the CO_2_ sensor detects a higher level of CO_2_ in the air (measured in ppm). The sensor used is sensitive enough to detect a single active individual in an enclosed OR with air circulation conditions of 20 air changes per hour (ACH) and positive pressure, which meets the ventilation requirements for an OR according to the American Society of Heating, Refrigeration and Air-Conditioning Engineers specification ASHRAE 170–2017. The performance demonstrated will facilitate selection of available technologies for use in future experiments and more complex systems for monitoring occupation level. The results provide fundamental data regarding CO_2_ accumulation in an OR that healthcare professionals can use to assess CO_2_ detection as a means to better understand and manage the occupation level and accumulative respiration in surgical environments. Awareness of occupation levels on air quality will encourage new and effective approaches to risk management strategies for minimizing surgical site infections. Note that while we focus on occupation level, it is also important to track the accumulation of respiratory byproducts which can contain harmful bioaerosols. While infectious agents might be difficult to detect, monitoring the changes in CO_2_ concentration provides a detectable proxy that can indicate if the byproducts of breathing are able to accumulate despite the presence of heavy air flow and positive pressure. We show that the CO_2_ level can accumulate as an individual breathes in an enclosed well-ventilated OR and that the CO_2_ concentration is dependent on the number of people in the room, which indicates that the relative contamination level of the air in an OR increases as people enter the room, even at standard OR ACH levels and positive pressure.

## Methods

Testing was performed in a 50 m^3^ operating room simulator, which included central ceiling-mounted ducts directing HEPA-filtered supply air and four lower wall mounted air return ducts. The system supplied 20 air exchanges per hour (ACH) and 0.03 in. H_2_O (7.5 Pa) of positive pressure to the outside environment. The room was equipped with typical OR air flow obstructions including surgical lights, tables and medical equipment (Fig. 1). Two calibrated Aeroqual 500 NDIR CO_2_ sensors (Gas Sensing) were used to measure the concentration of CO_2_. This sensing device can measure CO_2_ concentrations in the range of 0-5000 ppm with a resolution of 1 ppm. All measurements were performed at ambient temperature. Data was collected electronically and downloaded to an external computer for analysis using Aeroqual Series S500 Monitor Software V6.5 and Microsoft Excel. The sensors were placed in the perimeter of the OR because the perimeter is more contaminated than the center. The first sensor was placed approximately 58 cm from a wall containing the entry/exit door. The sensor was approximately 2.4 m from the door and approximately 1.8 m from the head of an operating bed in the middle of the room. The second sensor was placed approximately 58 cm from the opposite wall and approximately 2.4 m from the head of the operating bed and approximately 5.3 m from the door. The two sensors were approximately 4.6 m apart. Sensor readings were taken at 60 s intervals. For occupancy data, adults walked in the room for 20 minutes at a moderate pace in a circular path, changing direction every 5 minutes. The door was kept closed during the experiments. For occupancy data, the change in CO_2_ immediately before entering the room was compared to the CO_2_ concentration upon exit. For three-hour single occupancy experiments, an adult rested in a chair for the first hour of occupancy, walked in the room for 1 hour at a moderate pace in a circular path, changing direction every 5 minutes, and rested in the chair for the final hour.

## Results and discussion

**Fig. 2:**
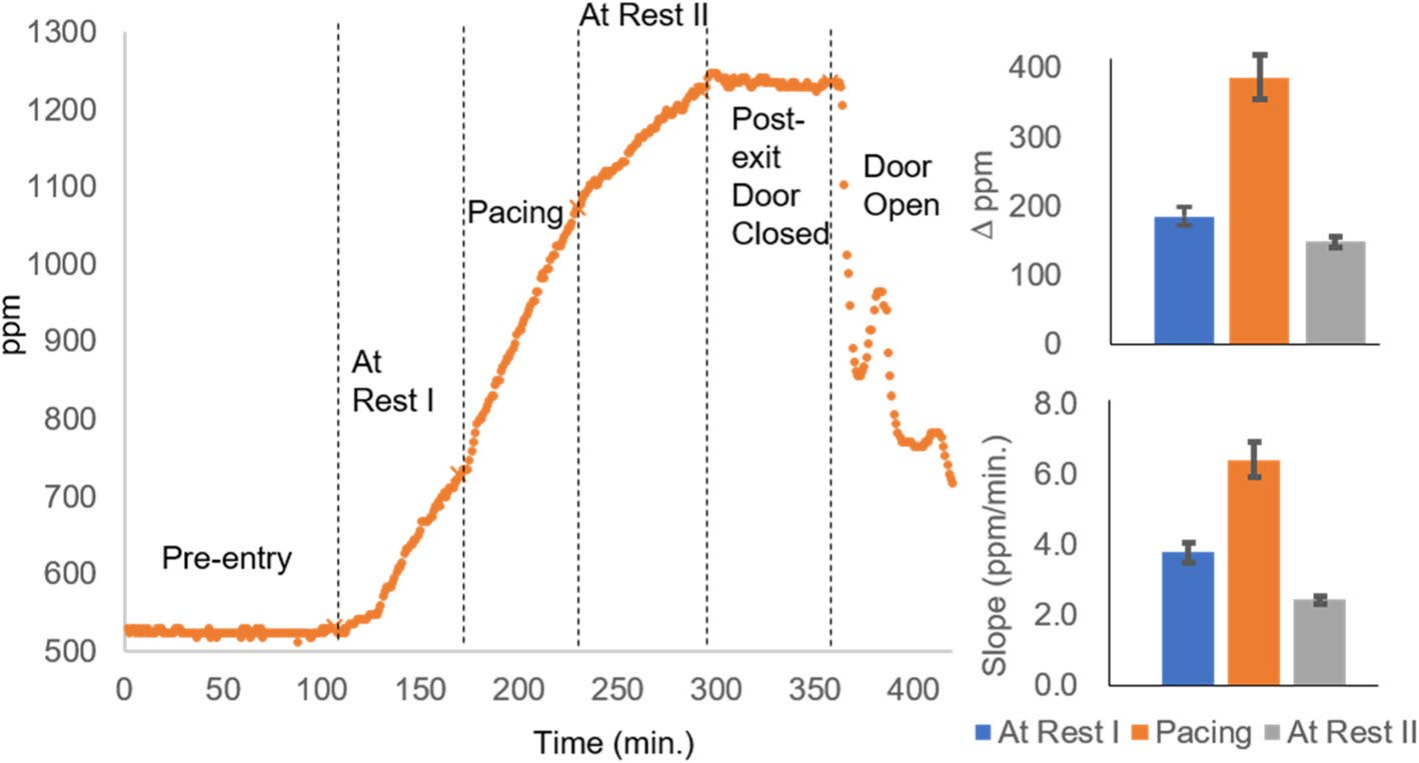
When an individual enters an enclosed room, the amount of detectable CO_2_ increases with occupation time. The rate of CO_2_ increase is dependent on the activity level of the individual. The CO_2_ increases more quickly when the individual paces the room compared to when the individual is at rest. The slopes are compared for the active and at rest conditions for the individual in the enclosed and non-ventilated room

In order to understand the sensitivity of our sensor to human respiration in an enclosed room, we initially monitored the change in CO_2_ concentration when one adult human resides in a simulated OR at ambient pressure and without ventilation. Two sensors were placed near opposite walls in the room in order to understand whether or not large spatiotemporal fluctuations in CO_2_ concentration develop. The CO_2_ level as a function of time for a representative trial is shown in Fig. 2 (sensor 2 data shown; see supporting information S1 for sensor 1 and subsequent trials data). Prior to entry, the background level of CO_2_ in the room was relatively constant, fluctuating around 525 ppm. After the individual entered the room and assumed a resting position seated in a chair, the CO_2_ level increased by approximately 200 ppm over a period of 1 hour. After 1 hour at rest, the individual stood up and walked in a circular path around the OR for 1 hour, reversing direction every 5 minutes. The increased activity level produced a faster rate of CO_2_ accumulation in the enclosed room, increasing by 350 ppm after 1 hour. After 1 hour of pacing the OR, the individual re-assumed the initial resting position. The CO_2_ level continued to rise, but at a reduced rate compared to the first and second hours in the OR, increasing by 156 ppm after 1 hour and bringing the total concentration to approximately 1259 ppm, which is approximately 2.4 times the initial background level. The individual then left the room, closing the door after exiting. The CO_2_ level remained relatively constant while uninhabited. After monitoring the CO_2_ level in the empty room for 1 hour, the door was propped open. The CO_2_ level rapidly reduced as the door remained open. Replication of the experiment multiple times showed consistency in the total change and rate of change in CO_2_ concentration for both sensors (See S1, S2, S3, S4 and S5).

The average change in CO_2_ concentration and average slope for each of the three hours spent in the room are shown in the bar graph on the right in Fig. 2. The rate of CO_2_ accumulation consistently increases when the individual transitions from a resting state to moderate activity, and consistently decreases when the individual re-assumes a resting state. The average slopes for the initial resting phase (At Rest I), the active phase (Pacing) and the second resting phase (At Rest II) are 3.8 ± 0.3 ppm/min., 6.4 ± 0.5 ppm/min. and 2.4 ± 0.1 ppm/min, respectively. The average change in CO_2_ for the corresponding phases are 190 ± 10 ppm, 390 ± 30 ppm and 150 ± 7 ppm, respectively. The results show that a single individual can considerably increase the concentration of CO_2_ in a room and, by extension, affect the quality of the air. Importantly, an individual’s contribution to the CO_2_ level in a room can be reliably detected with a portable NDIR sensor and the rate of increase corresponds to the occupant’s activity level. Higher activity increases metabolic rate which in turn increases CO_2_ production. A recent study has similarly concluded that measured CO_2_ generation rates are positively associated with physical activity levels [18]. In all trials the CO_2_ level did not exceed 1400 ppm, which is below the Occupational Safety and Health Administration (OSHA) permissible exposure limit of 5000 ppm (averaged over 8 hours), however, the need for awareness is apparent. As noted above, a growing body of cross-disciplinary literature in fields that include cognitive psychology, indoor air quality and neuroscience suggests that increasing CO_2_ concentrations can impair cognitive function. For example, Allen et al. [25] reported declines in “cognitive function scores” when indoor CO_2_ levels were increased from approximately 500 ppm to approximately 950 ppm, and greater declines when the CO_2_ level was raised to approximately 1400 ppm. Activities that involve increasing amounts of time and/or number of people in an enclosed space with limited ventilation could produce CO_2_ levels above permissible limits or exceed levels associated with compromised cognitive performance.

**Fig. 3:**
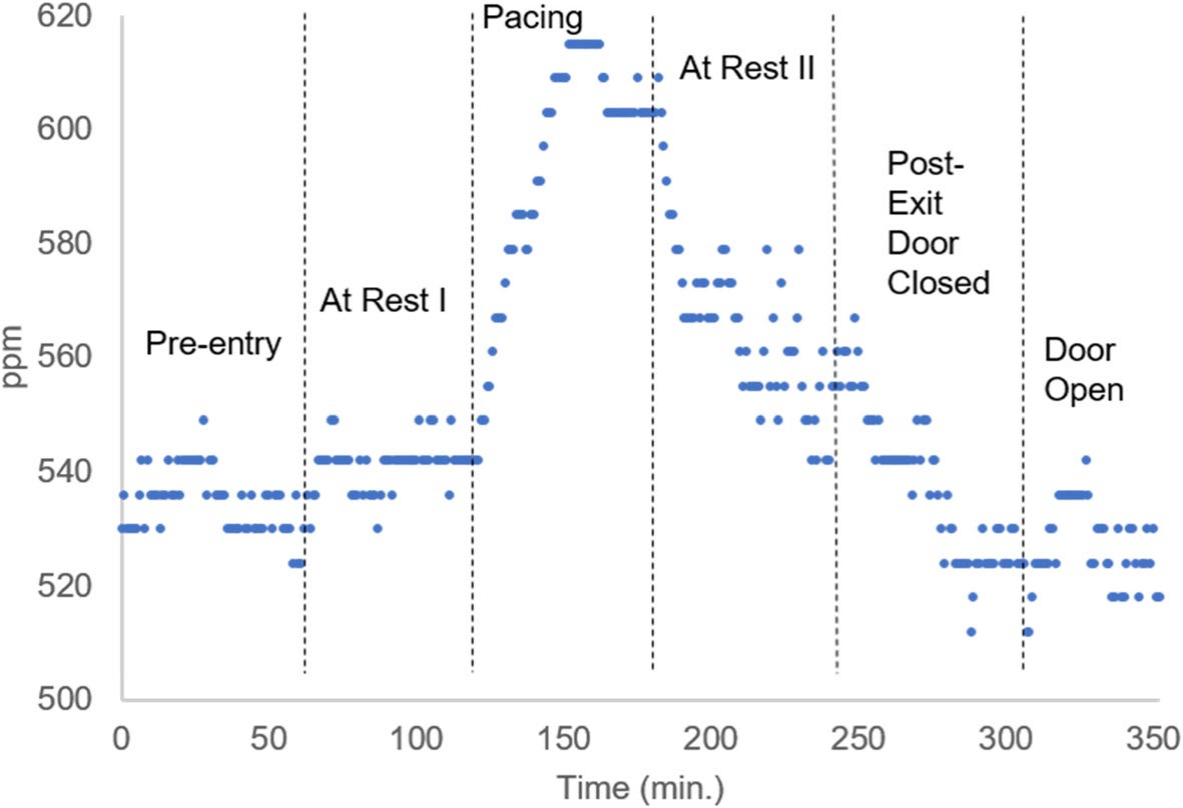
When an individual enters an enclosed room containing normal OR air flow conditions (positive pressure of 0.03 in. H_2_O and a ventilation system operating at 20 ACH), CO_2_ exhalation can be detected provided that the individual is moderately active. When a single person paces the OR for 1 h, the amount of CO_2_ in the room increases by up to approximately 73 ppm. When the active individual becomes seated and performs no physical activity the CO_2_ level decreases

In order to understand if metabolic CO_2_ production can be detected in an OR operating at standard conditions, we repeated the experiment under standard OR airflow conditions. The room ventilation operated at 20 air changes per hour (ACH) with a positive room pressure of 0.03 in. H_2_O. Figure 3 shows the CO_2_ concentration as a function of time for an individual in the enclosed OR. When at rest, it is very difficult to detect occupancy due to the air flow conditions. When the individual begins to walk in the room as described above, the CO_2_ level increases with time. After one hour of walking the maximum change detected was approximately 73 ppm. Similar results were obtained with the additional sensor in the room (see S6). This result shows that while the air circulation system and positive pressure have a beneficial effect on the air quality, it does not completely eliminate the respiratory influence on the air composition. The metabolic CO_2_ production of a single person can be detected even with standard OR air flow conditions, indicating that the exhalation products of one person can accumulate at a rate that is higher than its displacement by the ventilation system.

**Fig. 4:**
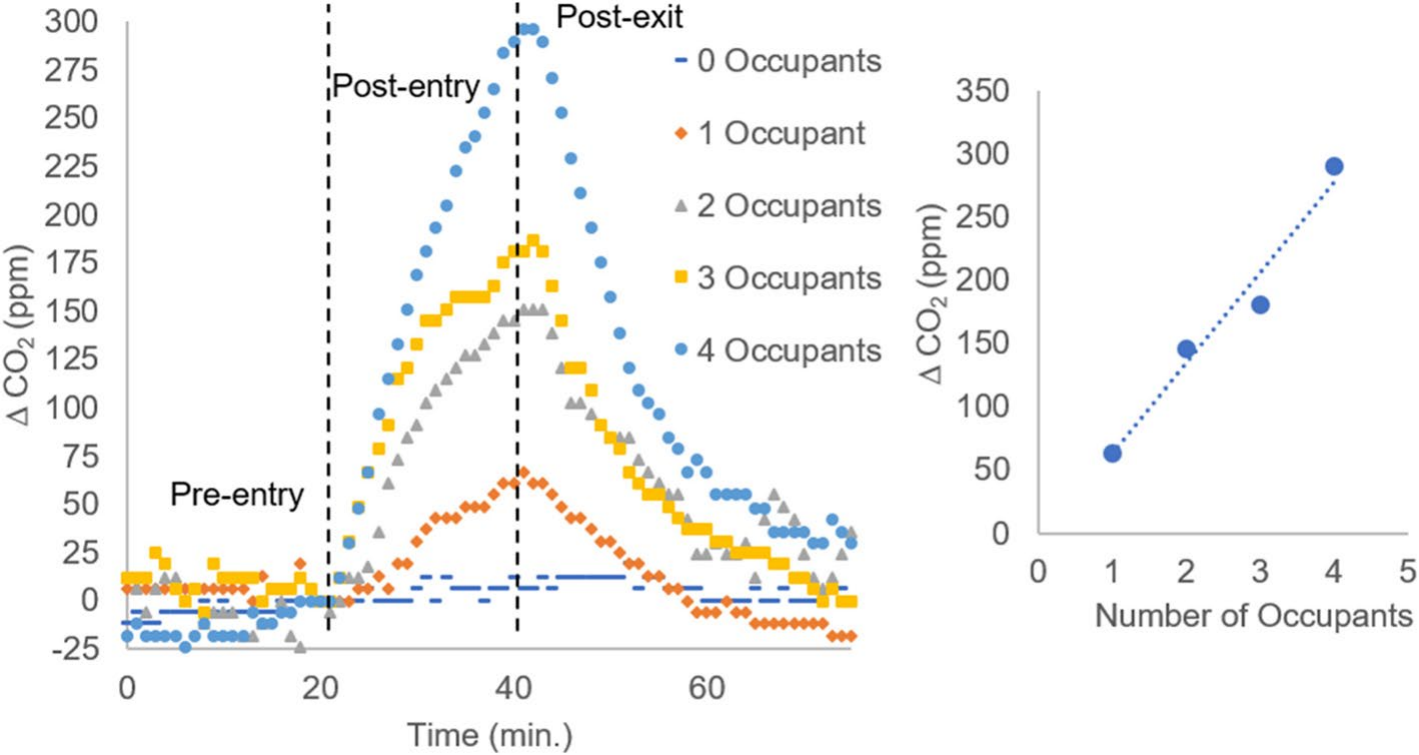
The amount of detected CO_2_ increases over a 20-minute period when individuals are present in an OR with an air circulation system operating at a level of approximately 20 ACH and a positive pressure of 0.03 in. H_2_O (left). As the number of individuals in the room increases, the amount of detected CO_2_ increases. Upon exit, the CO_2_ level rapidly decreases. A graph showing the change in the level of CO_2_ as a function of the number of occupants is presented on the right. Data shown are for sensor 2. See S7 for sensor 1 data

In order to understand if the CO_2_ level increases with the number of individuals in an OR, we monitored the CO_2_ level when occupied by a pre-selected number of individuals for a period of 20 minutes under normal OR air flow conditions. Figure 4 shows a graph of change in CO_2_ level as a function of time for different quantities of occupants. The presence of a single individual pacing the room produces a clear rise in CO_2_ concentration. After repeating the experiment three times the average increase for sensors 1 and 2 show accumulations of approximately 54 ± 7 ppm and 59 ± 9 ppm, respectively. After exiting and ensuring that the door was closed, the detected CO_2_ concentration briefly increases before showing a long-term attenuation to the baseline level as shown by the representative trace in Fig. 4. As the number of occupants in the room increases, the CO_2_ level increases. When four occupants pace the room for 20 minutes, the CO_2_ level increases by 290 ppm. When three and two occupants pace the room for 20 minutes, the respective CO_2_ concentrations are 181 and 145 ppm. A graph on the right in Fig. 4 displays the change in CO_2_ concentration as a function of the number of occupants that were in the room. Under the conditions employed, the CO_2_ rises in a linear fashion. These results show that as the number of people in a room increases, the CO_2_ concentration increases, even under air purifying ventilation standards that are encountered in an OR. Note that the levels of CO_2_ obtained will vary with individuals, activity levels and ventilation rates, however, our data shows a clear trend indicating that higher CO_2_ levels are detected when the number of occupants in a room increases. A difference of one person is enough to detect a change in CO_2_ concentration.

In order to minimize surgical site infections, it is important to maintain clean air in an OR. Individuals introduce a number of viable and non-viable impurities to the air in an OR through various mechanisms including shedding, displacing particulate matter at rest on a surface and breathing, the latter of which produces CO_2_ [34–37]. We have demonstrated that changes in CO_2_ concentration provide a biomarker of human presence that can be detected even under air flow conditions of 20 ACH and positive pressure. Tracking CO_2_ level in an OR can help manage human traffic and facilitate the epidemiology of surgical site infections. Although well-managed ORs are ventilated and in many cases under positive pressure, our results show that the presence of one breathing individual creates detectable changes in the composition of the air in a properly ventilated OR. While at relatively low concentrations CO_2_ itself is benign, it provides a proxy for more harmful substances that are emitted via expiration, including the highly disruptive SARS-CoV-2 which causes COVID-19 [23]. Additionally, a number of health effects associated with rising CO_2_ levels in an enclosed space include drowsiness, loss of concentration, nausea, headaches and more severe consequences associated with oxygen deprivation as higher concentrations are reached [38]. Gaining an awareness and understanding of the effect of occupancy time and population on the CO_2_ concentration in an OR will facilitate public health risk management and spur the development of new technologies for using CO_2_ to track the occupancy and respiratory history of an OR during surgery. In combination with good ventilation, particle filtration, PPE and UV disinfection, CO_2_ monitoring can be employed in the toolbox of techniques used to manage, assess and prevent hospital acquired infections.

## Conclusion

We have shown that the concentration of CO_2_ in an enclosed operating room increases as the room is populated. While the magnitude of increase is lower when the room is well-ventilated with positive pressure, the accumulation of CO_2_ over time reaches detectable levels and was shown to increase as more individuals occupied the room for a given amount of time. This study has important implications for developing systems for monitoring CO_2_ accumulation as well as building awareness for the involuntary and unavoidable contamination of the air in ORs upon human entry. Monitoring CO_2_ levels in an OR will provide critical information that can help mitigate risks that can lead to transmission of infections.

## Supplementary Information

**Additional file 1:** **S1. **Sensor 1, trial 1 (see Figure 1 for sensor 2 data): CO_2 _concentration as a function of occupation time for a single individual in an enclosed OR with no ventilation. **S2. **Sensor 2, trial 2: CO_2_ concentration as a function of occupation time for a single individual in an enclosed OR with no ventilation. **S3. **Sensor 1, Trial 2: CO_2_ concentration as a function of occupation time for a single individual in an enclosed OR with no ventilation. **S4. **Sensor 2, Trial 3: CO_2_ concentration as a function of occupation time for a single individual in an enclosed OR with no ventilation. **S5. **Sensor 1, Trial 3: CO_2_ concentration as a function of occupation time for a single individual in an enclosed OR with no ventilation. **S6. **Sensor 1: When an individual enters an enclosed room containing normal OR airflow conditions (positive pressure of 0.03 in. H_2_O and a ventilation system operating at 20 ACH), CO_2_ exhalation can be detected provided that the individual is moderately active. **S7. **Sensor 1 data: The amount of detected CO_2_ increases over a 20-minute period when individuals are present in an OR with an air circulation system operating at a level of approximately 20 ACH and a positive pressure of 0.03 in. H_2_O.

## Data Availability

All data generated and analyzed in this study are included in this publication and the supporting information file.

## Abbreviations

CO_2_: Carbon dioxide
OR: Operating room
SSI: Surgical site infection

## References

[1] Mandal J, Brandl H (2011). Bioaerosols in Indoor Environment - A Review with Special Reference to Residential and Occupational Locations. Open Environ Monit J.

[2] Napoli C, Marcotrigiano V, Montagna MT (2012). Air sampling procedures to evaluate microbial contamination: a comparison between active and passive methods in operating theatres. BMC Public Health.

[3] Chatoutsidou SE, Serfozo N, Glytsos T, Lazaridis M (2017). Multi-zone measurement of particle concentrations in a HVAC building with massive printer emissions: influence of human occupation and particle transport indoors. Air Qual Atmos Health.

[4] Pereira M, Tribess A, Buonanno G, Stabile L, Scungio M, Baffo I (2020). Particle and Carbon Dioxide Concentration Levels in a Surgical Room Conditioned with a Window/Wall Air-Conditioning System. Int J of Environ Res Public Health.

[5] Parvizi J, Barnes S, Shohat N, Edmiston CE (2017). Environment of care: Is it time to reassess microbial contamination of the operating room air as a risk factor for surgical site infection in total joint arthroplasty?. Am J Infect Control.

[6] Cook TM, Piatt CJ, Barnes S, Edmiston CE (2019). The Impact of Supplemental Intraoperative Air Decontamination on the Outcome of Total Joint Arthroplasty: A Pilot Analysis. J Arthoplasty.

[7] Kapadia BH, McElroy MJ, Issa K, Johnson AJ, Bozic KJ, Mont MA (2014). The economic impact of periprosthetic infections following total knee arthroplasty at a specialized tertiary-care center. J Arthoplasty.

[8] Obard JP, Fang LS (2003). Airborne Concentrations of Bacteria in a Hospital Environment in Singapore. Water Air Soil Pollut.

[9] Tang JW, Noakes CJ, Nielsen PV, Eames I, Nicolle A, Li Y, Settles GS (2011). Observing and quantifying airflows in the infection control of aerosol- and airborne-transmitted diseases: an overview of approaches. J Hosp Infect.

[10] Percival SL, Emanuel C, Cutting KF, Williams DW (2012). Microbiology of the skin and the role of biofilms in infection. Int Wound J.

[11] Wargocki P (2021). What we know and should know about ventilation. REHVA J..

[12] Turel I Occupant-generated CO_2_ as an Indicator of Ventilation Rate, Lawrence Berkeley National Laboratory, 1980, retrieved from https://escholarship.org/uc/item/70r2g8kq.

[13] Korsavi SS, Montazami A, Mumovic D (2020). Indoor Air Quality (IAQ) in Naturally- Ventilated Primary Schools in the UK: Occupant-Related Factors. Build Environ.

[14] Sakamoto M, Li M, Kuga K, Ito K, Beko G, Williams J, Wargocki P (2021). CO2 emission rates from sedentary subjects under controlled laboratory conditions. Build Environ.

[15] Qi MW, Li XF, Weschler LB, Sundell J (2014). CO_2_ generation rate in Chinese people. Indoor Air.

[16] Kuga K, Ito K, Wargocki P (2021). The effects of warmth and CO2 concentration, with and without bioeffluents, on the emission of CO2 by occupants and physiological responses. Indoor Air.

[17] Zhai Y, Li M, Gao S, Yang L, Zhang H, Arens E, Gao Y (2018). Indirect calorimetry on the metabolic rate of sitting, standing and walking office activities. Build Environ.

[18] Yang L, Wang X, Li M, Zhou X, Liu S, Zhang H, Arens E, Zhai Y (2020). Carbon dioxide generation rates of different age and gender under various activity levels. Build Environ.

[19] Gall ET, Mishra AK, Li J, Schiavon S, Laguerre A (2020). Impact of cognitive tasks on CO2 and isoprene emissions from humans. Environ Sci Technol.

[20] Nomoto A, Hisayama R, Yoda S, Akimoto M, Ogata M, Tsutsumi H, Tanabe S (2021). Indirect calorimetry of metabolic rate in college-age Japanese subjects during various office activities. Build Environ.

[21] Persily A, de Jonge L (2017). Carbon dioxide generation rates for building occupants. Indoor Air.

[22] Rudnick SN, Milton DK (2003). Risk of indoor airborne infection transmission estimated from carbon dioxide concentration. Indoor Air.

[23] Peng Z, Jimenez JL (2021). Exhaled CO_2_ as a Covid-19 Infection Risk Proxy for Different Indoor Environments and Activities. Environ Sci Technol Lett.

[24] Tortora GJ, Anagnostakos NP (1981). Principles of Anatomy and Physiology.

[25] Allen JG, MacNaughton P, Satish U, Santanam S, Vallarino J, Spengler JD (2016). Associations of cognitive function scores with carbon dioxide, ventilation, and volatile organic compound exposures in office workers: A controlled exposure study of green and conventional office environments. Environ Health Perspect.

[26] Harter M (1967). Effects of carbon dioxide on the alpha frequency and reaction time in humans. Electroencephalogr Clin Neurophysiol.

[27] Sayers JA, Smith RE, Holland RL, Keatinge WR (1987). Effects of carbon dioxide on mental performance. J Appl Physiol.

[28] Wargocki P, Porras-Salazar JA, Contreras‐Espinoza S, Bahnfleth W (2020). The relationships between classroom air quality and children’s performance in school. Build Environ.

[29] Du B, Tandoc MC, Mack ML, Siegel JA (2020). Indoor CO_2_ concentrations and cognitive function: A critical review. Indoor Air.

[30] Karthikeyan CP, Samuel AA (2008). CO_2_ dispersion studies in an operating theatre under transient conditions. Energy Build.

[31] Martin CR, Zeng N, Karion A, Dickerson RR, Ren X, Turpie BN, Weber KJ (2017). Evaluation and environmental correction of ambient CO_2_ measurements from a low-cost NDIR sensor. Atmos Meas Tech.

[32] Yasuda T, Yonemura S, Tani A (2012). Comparison of the Characteristics of Small Commercial NDIR CO2 Sensor Models and Development of a Portable CO2 Measurement Device. Sensors.

[33] Fine GF, Cavanagh LM, Afonja A, Binions R (2010). Metal Oxide Semi-Conductor Gas Sensors in Environmental Monitoring. Sensors.

[34] Noble WC, Habbema JDF, van Furth R, Smith I, de Raay C (1976). Quantitative studies on the dispersal of skin bacteria into the air. J Med Microbiol.

[35] Ferro AR, Kopperud RJ, Hildemann LM (2004). Source strengths for indoor human activities that resuspend particulate matter. Environ Sci Technol.

[36] Johnson GR, Morawska L (2009). The mechanism of breath aerosol formation. J Aerosol Med Pulm Drug Deliv.

[37] Nicas M, Nazaroff WW, Hubbard A (2005). Toward Understanding the Risk of Secondary Airborne Infection: Emission of Respirable Pathogens. J Occup Environ Hyg.

[38] Wisconsin Department of Health Services from. https://www.dhs.wisconsin.gov/chemical/carbondioxide.htm.

