# Supplementary material for: Increased CO2 levels in the operating room correlate with the number of healthcare workers present: an imperative for intentional crowd control
Source: Patient Saf Surg. 2022 Nov 17;16:35. doi: 10.1186/s13037-022-00343-8 (PMC9672642; doi:10.1186/s13037-022-00343-8)
Supplement: Supplementary file 1 — Additional file 1: S1. Sensor 1, trial 1 (see Figure 1 for sensor 2 data): CO2 concentration as a function of occupation time for a single individual in an enclosed OR with no ventilation. S2. Sensor 2, trial 2: CO2 concentration as a function of occupation time for a single individual in an enclosed OR with no ventilation. S3. Sensor 1, Trial 2: CO2 concentration as a function of occupation time for a single individual in an enclosed OR with no ventilation. S4. Sensor 2, Trial 3: CO2 concentration as a function of occupation time for a single individual in an enclosed OR with no ventilation. S5. Sensor 1, Trial 3: CO2 concentration as a function of occupation time for a single individual in an enclosed OR with no ventilation. S6. Sensor 1: When an individual enters an enclosed room containing normal OR airflow conditions (positive pressure of 0.03 in. H2O and a ventilation system operating at 20 ACH), CO2 exhalation can be detected provided that the individual is moderately active. S7. Sensor 1 data: The amount of detected CO2 increases over a 20-minute period when individuals are present in an OR with an air circulation system operating at a level of approximately 20 ACH and a positive pressure of 0.03 in. H2O. [file 13037_2022_343_MOESM1_ESM.docx]

**Supporting Information**

**Increasing Occupancy Levels in an Operating Room Environment Increases Detectable Ambient CO_2_ Concentrations**


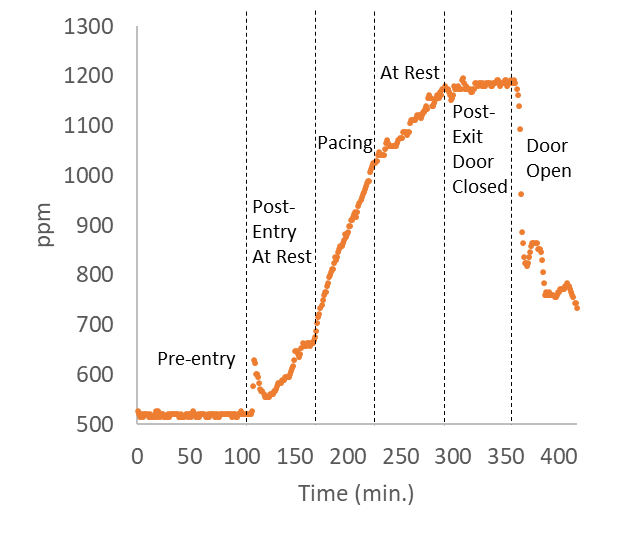


S1: Sensor 1, trial 1 (see Figure 1 for sensor 2 data): CO_2_ concentration as a function of occupation time for a single individual in an enclosed OR with no ventilation. When an individual enters an enclosed room, the amount of detectable CO_2_ increases with occupation time. The rate of CO_2_ increase is dependent on the activity level of the individual. The CO_2_ increases more quickly when the individual paces the room compared to when the individual is at rest.


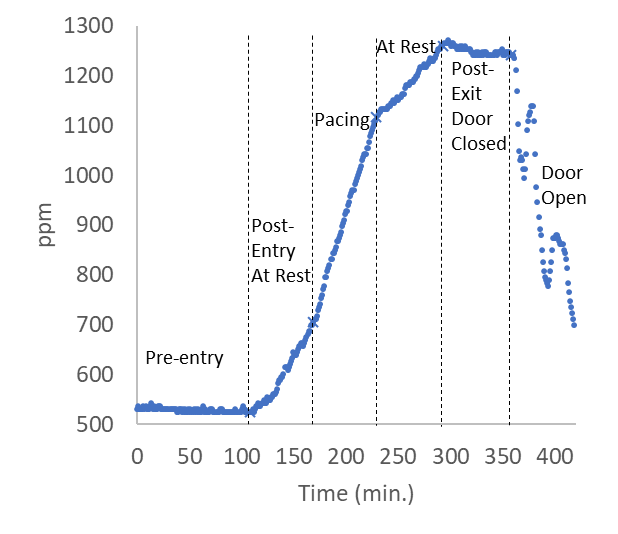


S2: Sensor 2, trial 2: CO_2_ concentration as a function of occupation time for a single individual in an enclosed OR with no ventilation. When an individual enters an enclosed room, the amount of detectable CO_2_ increases with occupation time. The rate of CO_2_ increase is dependent on the activity level of the individual. The CO_2_ increases more quickly when the individual paces the room compared to when the individual is at rest.


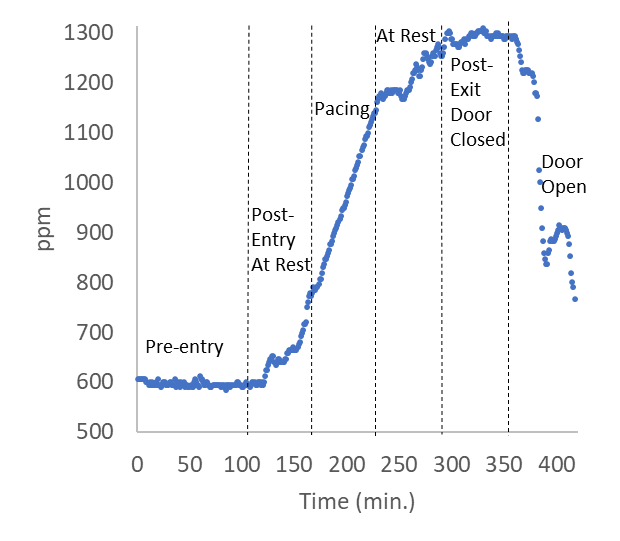


S3: Sensor 1, Trial 2: CO_2_ concentration as a function of occupation time for a single individual in an enclosed OR with no ventilation. When an individual enters an enclosed room, the amount of detectable CO_2_ increases with occupation time. The rate of CO_2_ increase is dependent on the activity level of the individual. The CO_2_ increases more quickly when the individual paces the room compared to when the individual is at rest.


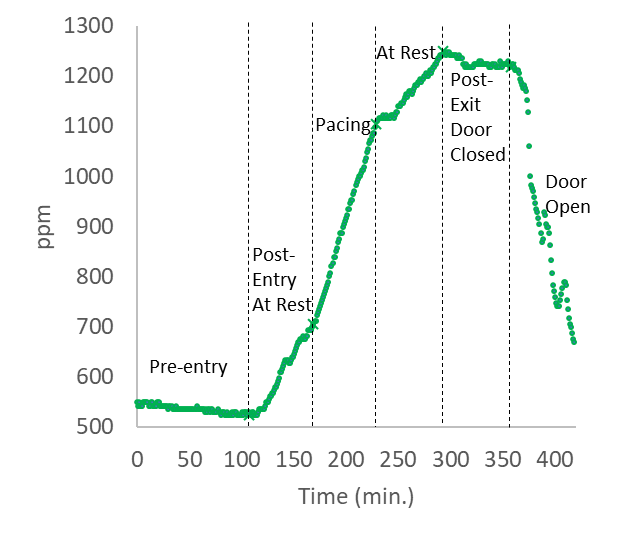


S4: Sensor 2, Trial 3: CO_2_ concentration as a function of occupation time for a single individual in an enclosed OR with no ventilation. When an individual enters an enclosed room, the amount of detectable CO_2_ increases with occupation time. The rate of CO_2_ increase is dependent on the activity level of the individual. The CO_2_ increases more quickly when the individual paces the room compared to when the individual is at rest.


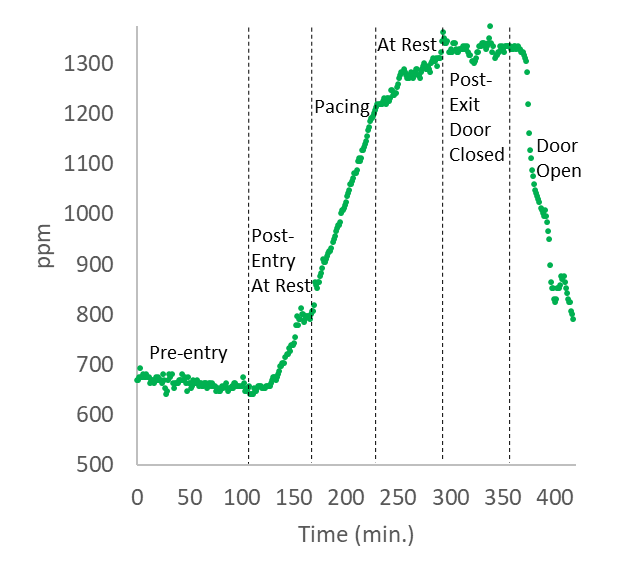


S5: Sensor 1, Trial 3: CO_2_ concentration as a function of occupation time for a single individual in an enclosed OR with no ventilation. When an individual enters an enclosed room, the amount of detectable CO_2_ increases with occupation time. The rate of CO_2_ increase is dependent on the activity level of the individual. The CO_2_ increases more quickly when the individual paces the room compared to when the individual is at rest.


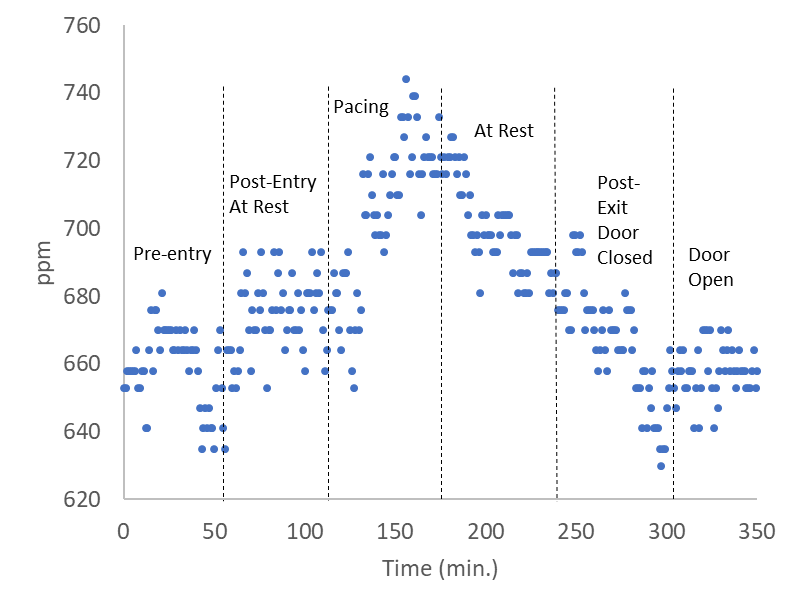


S6: Sensor 1: When an individual enters an enclosed room containing normal OR air flow conditions (positive pressure of 0.03 in. H_2_O and a ventilation system operating at 20 ACH), CO_2_ exhalation can be detected provided that the individual is moderately active. When a single person paces the OR for 1 hour, the amount of CO_2_ in the room increases. When the active individual becomes seated and performs no physical activity the CO_2_ level decreases.


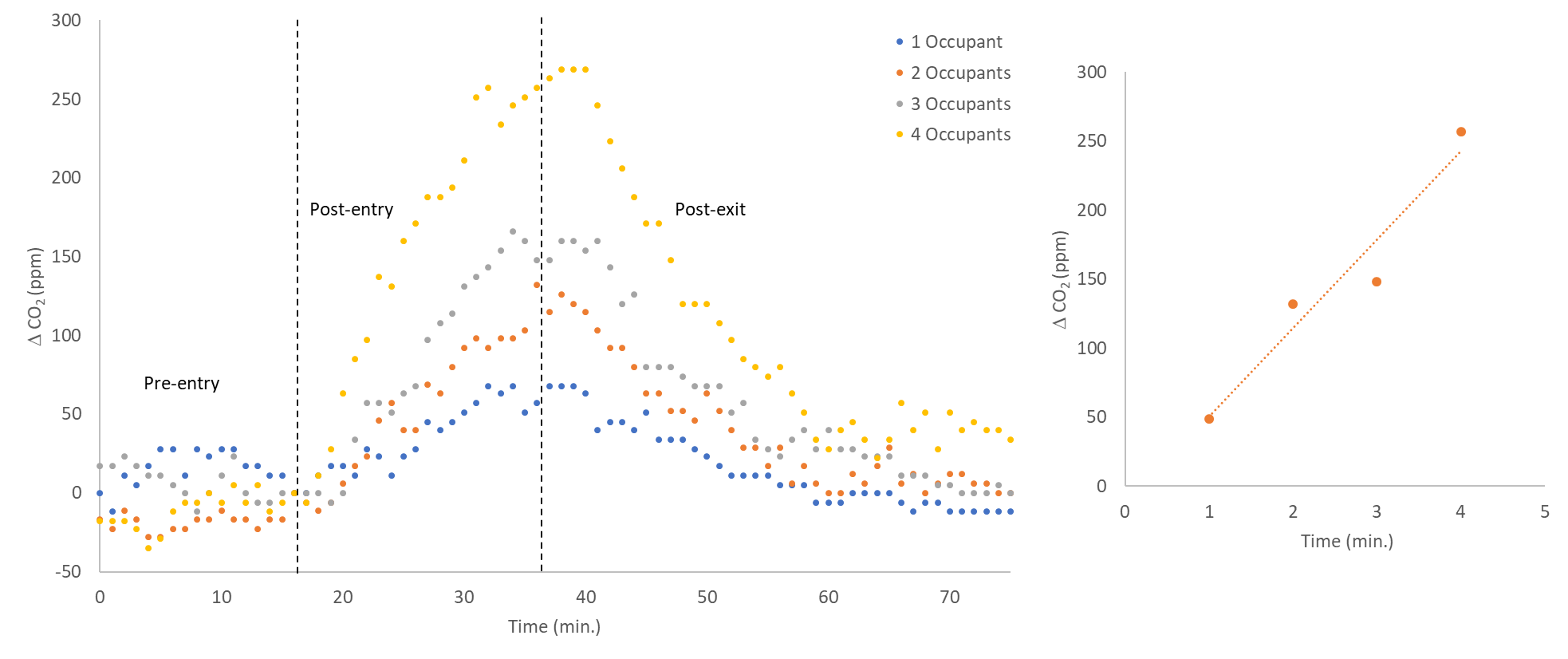


S7: Sensor 1 data: The amount of detected CO_2_ increases over a 20-minute period when individuals are present in an OR with an air circulation system operating at a level of approximately 20 ACH and a positive pressure of 0.03 in. H_2_O. As the number of individuals in the room increases, the amount of detected CO_2_ increases. Upon exit, the CO_2_ level decreases.
